# Live porcine thirty days delayed recovery surgery: Qualitative findings with the hypersonic vitrectomy

**DOI:** 10.1371/journal.pone.0197038

**Published:** 2018-06-01

**Authors:** Soon Wai Ch’ng, Luciane Dreher Irion, Richard Bonshek, Joseph Shaw, Alessandro Papayannis, Salvador Pastor-Idoate, Paulo Eduardo Stanga

**Affiliations:** 1 Manchester Royal Eye Hospital, Manchester University NHS Foundation Trust, Manchester Academic Health Science Centre, Manchester, United Kingdom; 2 Manchester Vision Regeneration Lab, Manchester University NHS Foundation Trust, Manchester Academic Health Science Centre, Manchester, United Kingdom; 3 Histopathology Department / National Specialist Ophthalmic Pathology Service, Manchester University NHS Foundation Trust, Manchester, United Kingdom; 4 Division of Evolution and Genomic Sciences, School of Biological Sciences, Faculty of Biology, Medicine and Health, University of Manchester, Manchester Academic Health Science Centre, Manchester, United Kingdom; Massachusetts Eye & Ear Infirmary, Harvard Medical School, UNITED STATES

## Abstract

**Introduction:**

Qualitatively assess the possible delayed structural, macroscopic and microscopic changes in the neuro-retina, retinal vasculature, retinal pigment epithelium (RPE) and optic nerve head (ONH) after pars plana vitrectomy (PPV) surgery using a new hypersonic vitrector (HV).

**Materials and methods:**

Eight live porcine eyes underwent PPV using either the HV or a conventional pneumatic guillotine vitrector (GV). The un-operated fellow eye from each pig was used as an external control. The pigs were post-operatively kept alive for 30 days before termination and enucleation of the eyes. Prior to enucleation, all eyes underwent examination of the lens and indirect ophthalmoscopy. Enucleated eyes were fixed in formalin, examined macroscopically and processed for histological assessment. Microscopic analysis included assessment of neuro-retina, retinal vasculature, RPE and ONH, as well as observation for any morphological intraocular changes. Comparison was made between: (1) treated and untreated areas of the same eye (internal control) (2) different areas within the same eye operated on using different vitrector settings (3) eyes operated on with the HV and GV (4) eyes receiving surgery and the fellow un-operated eye (external control, same pig).

**Results:**

All lenses had remained clear at 30 days into the postoperative period. On indirect ophthalmoscopy, the retina had remained attached in all eyes with no visible changes to the neuro-retina, retinal vasculature, RPE or ONH. Two eyes showed localized RPE depigmentation secondary to previously documented intraoperative retinal touch. The Morphological changes in the retinal layers above depigmented RPE were no different from retinal change elsewhere. There were mild and similar microscopic changes observed in the neuro-retina, retinal vasculature, RPE or ONH associated with either the HV or GV PPVs. Preliminary histological findings revealed no significant differences between eyes operated on with the HV and those operated on the GV.

**Discussion:**

At 30 days into the postoperative period, there seemed to be similar morphological changes attributable to the use of HV and GV. Therefore, the HV promises to be a new alternative to the currently commercially available GV for PPV.

## Introduction

The 23-gauge (23G) hypersonic vitrectomy (HV) (Vitesse^®^, Bausch+Lomb (B+L), St. Louis, USA) is a new capability that is being developed by B+L. The HV feature includes the introduction of a novel vitrectomy hand piece that uses a very small stroke length (0 to 60μm) ultrasonic (US) needle tip motion to generate oscillating high speed flow near the port to liquefy the vitreous at the tip and remove it from the posterior segment of the eye with minimal traction to the vitreous. The HV needle employs a closed, single-needle design with a small continuously-open port allowing smaller port size and larger inner-lumen diameters. This lowers the flow resistance and required infusion pressures. This effectively gives the tip of the HV to oscillate at 1.7 million cycles per minute which effectively “hypersonic liquefy” instead of cutting the vitreous. Current vitrectomy systems employ an inner/outer needle design where the inner needle is actuated pneumatically resulting in a guillotine-like cutting action at the interface between the inner needle and outer needle port. Unlike HV, the pneumatic guillotine vitrectomy (GV) needle is not continuously open but is interrupted by the guillotine cutting acting of the inner needle, resulting in discontinuous flow rate that may generate greater vitreous traction [1].

This study is the follow up to the ultrastructural and histopathological findings after pars plana vitrectomy (PPV) in cadaveric porcine and human eyes and non-delay live porcine eyes. In our previous study, light microscopy and electron microscopy showed similar morphological changes in the retina, vitreous and crystalline lens after PPV [2]. The HV have also produced consistent water and vitreous flow rates across the range of stroke length and aspiration levels [3]. Therefore, the aim of this study is to qualitatively compare the safety and efficacy using the HV and GV during PPV in 30 days delayed recovery of porcine eyes.

## Material and methods

This is a prospective observational study comparing the HV (test device) and GV (control device) to determine if there are any delayed or lingering effects of vitrectomy surgery observable 30 days following the procedure. This study was approved by the Research and Ethics Committee of the Animal Welfare and Ethical Review Body of the University of Manchester (UK) and the Home Office inspector (PPL-70/8382), The study was conducted according to the principles of the Animal Scientific Procedures 1986 Act, Ec86/609 Directive 2010/63/EU, and adhered to the Association for Research in Vision and Ophthalmology statement for the use of animals in ophthalmic and vision research.

The primary objective was to determine if there are any subjective morphological changes on the retina (posterior and peripheral) at a macro- or microscopic level 30 days following PPV using HV and GV, with preset vitrectomy settings and techniques. Another primary objective was to determine if there are any differences in retinal impact 30 days following the PPV, when the HV is operated near the retina (1mm) at various stroke length settings. The secondary objective was assessing the effectiveness of the HV (225μm diameter round needle port needle) in inducing posterior vitreous detachment (PVD). Another secondary objective was to assess and compare the efficiency of the HV and GV for core vitrectomy. The surgeon’s subjective assessment of the device and time to perform each procedure was also recorded.

### Preoperative, intraoperative and postoperative procedure

Sixteen eyes from eight 20-week-old Landrace commercial swine (Centre for Integrative Mam- malian Biology, Faculty of Life Sciences, University of Manchester, Manchester, UK) weighing 15–20 kg were used in the study. On the day prior to surgery, each animal was weighed, the body weight recorded and food was withheld overnight. On the day of surgery, an initial topical dose of tropicamide 1.0% and phenylephrine 2.5% was instilled in the eyes for dilation. Instillation of the same drops was re-applied at approximate 10-minute intervals to achieve maximum dilation of the iris. Surgery was initiated once a sufficient dilation of the eye was achieved. General anaesthesia was induced with an intramuscular injection of a combination tiletamine/zolazepam (Telazol^®^) at 4.4 mg/kg and xylazine at 2.2 mg/kg (prepared combination dosed at 0.044 ml/kg). When a sufficient level of anaesthesia was reached, an endotracheal tube was placed, then the animal was attached to an anaesthesia machine. The animal was maintained on an isoflurane anaesthesia for the remainder of the preparation and surgical procedure. An intravenous (IV) catheter was placed in a peripheral ear vein for fluid administration. IV fluids were administered at a rate of 10 ml/kg/hr during the surgical procedure. The peri-orbital area was shaved and a periocular surgical prep was performed by swabbing the area using a 1:50 dilution of povidone iodine in saline. The pigs were draped in a sterile manner and positioned under the operating microscope. Topical anaesthetic proparacaine hydrochloride (0.5%) was instilled into the draped eyes. A wire lid speculum was used to retract the eyelids. The pigs were anesthetized with a mixture of 4% isoflurane on 100% oxygen via face mask, intubated and maintained on isoflurane and 100% oxygen. The concentration of isoflurane was the lowest required to produce full surgical anaesthesia (between 2.5% and 3%). Levels of anaesthesia and animal welfare were monitored via capnography and pulse oximetry.

Three port 23G PPV surgery was performed. The vitrector needles were introduced into the posterior segment of the eye through the sclera via a trans-scleral entry site alignment (ESA) cannula. Surgeries were performed using the B+L StellarisPC Vision Enhancement system with hardware and software modifications to provide HV drive capability (Bausch+Lomb, St. Louis, USA). ESAs were inserted using auto-inserters or manual trocars through the sclera, 4 mm from the limbus, approximately three clock-hours apart. One port was used for the infusion cannula connected to a sterile bottle of Balanced Salt Solution, the second port for the vitrector and the third for the illumination probe. A wide-field fundus non-contact lens and clear gel on the cornea were used to aid in visualizing the peripheral retina. Procedures were conducted with the aid of a Leica M822 F20 (Leica Microsystems, Wetzlar, Germany) operating microscope. The microscope was used to visualize the ocular structures and allow assessment of the potential differences in ocular findings between the two devices.

All vitrectomy procedures were performed by a single surgeon (PES) and assisted by his clinical research fellow (SWC). The details of each surgical procedure are included in Table 1. Following the surgical procedures, the animals were kept alive for 30 days. During this period the animals were given a topical treatment (prednisolone acetate 1% 4x/day, cyclopentolate 1.0% 2x/day and chloramphenicol 0.5% 4x/day) each day. The pigs were monitored daily by the faculty member of the Centre for Integrative Mammalian Biology. PES or SWC examined the pigs on Day 1 post-operative and each week until the day of enucleation. The pig eyes were examined by the study team on the first postoperative day, and then weekly. After 30 days, treated eyes were given a final examination and then both eyes were gently enucleated and extraocular tissue gently removed from each globe. Following enucleation, and while under anesthesia, the animals were euthanized with pentobarbitone (150 mg/kg) by intra-cardiac puncture. The Named Veterinary Surgeon was available during the conduct of the procedures to assist with or act as a consultant for veterinary and animal welfare issues.

**Table 1:** Surgical procedures and system settings for operated eyes.

Eye No.	Hand piece / Procedure	Procedure Steps	Stroke Length for HV (μm) / CPM for GV	Vacuum rate (mm Hg)	Infusion pressure (mm Hg)
1^a^	**23G HV (175 μm port diameter) with full vitrectomy and no PVD**	Core vitreous removal	40	0–150	45
		Posterior vitreous removal–No PVD induced	N/P	N/P	N/P
		Peripheral vitreous removal	N/P	N/P	N/P
3	**23G HV (175 μm port diameter) with full vitrectomy and no PVD**	Core vitreous removal	40	0–150	45
		Posterior vitreous removal–No PVD induced	10–16	0–100	40
		Peripheral vitreous removal	10–20	0–100	40
5	**23G HV (225 μm port diameter) with full vitrectomy and PVD**	Core vitreous removal	40	0–150	55
		PVD inducement	0	0–600	55
		Posterior vitreous removal	10–16	0–100	50
		Peripheral vitreous removal	N/P	N/P	N/P
7	**23G HV (225 μm port diameter) with full vitrectomy and PVD**	Core vitreous removal	40	0–150	55
		PVD inducement	0	0–600	55
		Posterior vitreous removal	N/P	N/P	N/P
		Peripheral vitreous removal	N/P	N/P	N/P
9	**23G GV with full vitrectomy and PVD**	Core vitreous removal	4000	350–400	35
		PVD inducement	0	0–600	40
		Posterior vitreous removal	5000	0–300	40
		Peripheral vitreous removal	5000	0–300	40
11	**23G HV (225 μm port diameter) with full vitrectomy and PVD**	Core vitreous removal	40	0–150	45
		PVD inducement	0	0–600	45
		Posterior vitreous removal	10–40	0–100	45
		Peripheral vitreous removal	10–16	0–100	45
13	**23G GV with full vitrectomy and no PVD**	Core vitreous removal	4000	350–400	30
		Posterior vitreous removal–No PVD induced	5000	0–300	30
		Peripheral vitreous removal	5000	0–300	30
15	**23G HV (225 μm port diameter) with various stroke length settings near retina for 1 minute**	Area 1 –Nasal macula	10	10	30
		Area 2 –Temporal macula	20	10	30
		Area 3 –Superior to the optic nerve head, 2 disc diameters away from the macula	30	10	30
		Area 4 –Inferior to the optic nerve head, 2 disc diameters away from the macula	40	10	30

^a^Animal subject (Eye no.1) died immediately from cardiac arrest follow surgery and therefore eye samples not available for assessment.

CPM: cuts per minute; GV: guillotine vitrectomy; HV: hypersonic vitrectomy; N/P: Not performed; PVD: posterior vitreous detachment

#### Macroscopic and microscopic assessment methods

Eyes were assessed intraoperatively, with ocular clinical examination using the indirect ophthalmoscope and 20 diopters lens prior to enucleation, and postoperatively (macro- and microscopic) to evaluate performance against the study objectives. During the surgical procedures, intraoperative device performance and eye tissue impact were subjectively assessed by the surgeon and study team, and recorded by sponsor personnel. Following the surgical procedure, all animals were kept alive for 30 days. After the 30 days, all eyes receiving treatment were assessed via ocular clinical examination, and then all eyes were enucleated. Following the planned procedures, both eyes were gently enucleated and extraocular tissue gently removed from each globe. The animals were euthanized following enucleation.

The globes were kept separate and identified by pig number and as right or left eye. Each globe was transferred into a container filled with 10% neutral buffered formalin (NBF) for fixation until trimming. Each globe was photographed macroscopically (MacroPATH, Milestone Srl, Italy) prior to dissection. Limbal area was inked (3 o’clock on right eyes and 9 o’clock on left eyes) to facilitate histological orientation. The globe was trimmed in a manner that allowed the areas of designated interest to be incorporated into histologic sections. This involved cutting the globe along a horizontal plane superior to the cornea and optic nerve followed by a parallel cut inferior to the cornea and optic nerve. Each horizontal slice was placed in a cassette of appropriate size to accommodate the section of ocular tissue with temporal portion longer than the nasal portion. After processing the samples were embedded in paraffin wax blocks. Remainder calottes of each globe not processed were saved in 10% NBF. Three tissue sections of approximately 4μm in thickness were prepared from the paraffin blocks at five different levels. Each block was faced until a tissue section appeared, then the block was sectioned to a depth of 50μm. The block was then sectioned to the location of the optic disc and tissue sections were obtained. Additional tissue sections were obtained at three more levels, each at additional step depths of 1mm. Three additional sections were obtained at steps of 50μm. Histological sections were stained with hematoxylin and eosin to evaluate the potential ocular changes. In addition, a section at the level of the optic disc was stained with periodic acid-Schiff stain as well. These sections were examined microscopically and a description of the microscopic findings was recorded. All slides were evaluated in a masked fashion for the type of cutter and settings by two histopathologists (LDI, RB) separately using a microscope (Olympus BX51, Olympus Optical Co, Japan). Microscopic features were photographed using a camera (Olympus U-CMAD3, Japan) and CellD imaging software (Olympus Soft Imaging Solutions GMBH, Germany).

## Results

The first pig (Eyes No.1 and 2) died following the surgery from cardiac arrest and the ocular specimen was not taken for assessment. Eyes No.3, 5, 7, 11 and 15 underwent HV, Eyes No.9 and 13 underwent GV and Eyes No. 4, 6, 8, 10, 12, 14 and 16 were the control samples. Eye No.15 underwent surgery involving use of various stroke length settings near different areas of the retina to investigate the differential effects of stroke length. Table 2 provides a summary of reported findings from the pre-enucleation ocular clinical exams, and post-enucleation macroscopic and microscopic assessments by vitrector type. The control samples are also included for comparative purposes.

**Table 2:** Summary of histopathological findings of the eyes for the HV Device, GV and control samples. (Eye No.15 was excluded in this table as the subject was tested only for effects of different stroke length which differs from other eyes).

Finding	Exam Type	23G HV				23G GV		Control Samples						
		3	5	7	11	9	13	4	6	8	10	12	14	16
***Conjunctiva***														
**Congested**	Macroscopic	N	N	N	N	N	N	N	N	N	M	N	N	N
**Peri-limbal pigmentation**	Macroscopic	N	N	N	N	N	N	N	N	N	N	N	M	N
***Sclera***														
**Congested**	Macroscopic	N	V	D	M	N	N	N	N	N	N	N	N	D
**Irregular depigmentation -on**	Macroscopic	N	N	N	N	M	M	N	N	N	M	N	M	N
***Vitreous***														
**RBCs ± pigment granules**	Microscopic	N	N	N	N	N	N	N	N	N	N	A	N	N
***Retina***														
**Discoloration (patchy, paleness)**	Macroscopic	V	V	V	V	N	N	N	N	N	N	N	N	N
**RD (small ± artefactual)**	Macroscopic	N	A	A	A	N	A	A	A	A	A	N	A	N
**Folds**	Macroscopic	N	N	N	N	N	N	N	N	N	A	A	N	A
**Vacuolation (GCL, NFL, ± INL)**	Microscopic	M	D	M	V	M	V	D	D	D	D	M	M	D
**Thinning**	Microscopic	N	M	M	N	N	N	N	N	N	N	V	N	V
**Disruption of INL**	Microscopic	N	N	N	A	N	N	N	N	N	N	N	N	N
***Optic nerve***														
**Blurred**	Macroscopic	A	N	N	N	N	N	N	N	N	N	N	N	N
***RPE***														
**Decreased pigmentation**	Microscopic	M	N	M	N	N	N	N	N	N	N	N	N	N
**Atrophy**	Clinical Exam	N	D^a^	N	N	N	N	N	N	N	N	N	N	N
**Mild change**	Clinical Exam	N	N	N	N	N	V	N	N	N	N	N	N	N

^a^Likely caused by retinal touch noted during surgery

N = No changes, A = Artefactual, V = Very Minor, M = Minor, D = Moderate and S = Severe

GCL: Ganglion cell layer; ONL: outer nuclear layer; INL: inner nuclear layer; RBC: red blood cell; RD: retinal detachment; RPE: retinal pigment epithelium

**Fig 1:**
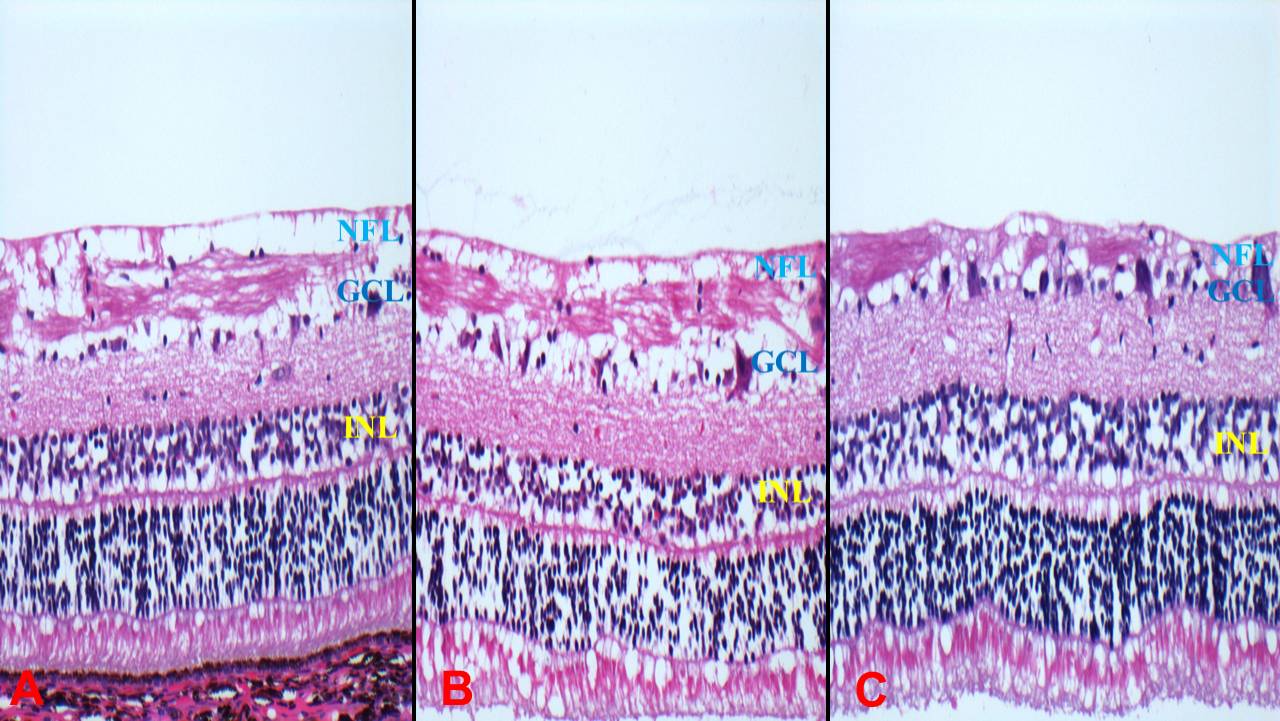
Haematoxylin-eosin stained horizontal retinal sections from porcine enucleated eyes after HV and GV PPV and control samples. (A) Retinal section from a control eye without any procedure (B) Retinal sections from eye that underwent HV PPV (C) Retinal sections from eye that underwent GV PPV. Cytoplasmic vacuolation and disruption in GCL, INL and NFL (A,B,C). (GCL: ganglion cell layer; GV: guillotine vitrector; HV: hypersonic vitrector; INL: inner nuclear layer; NFL: nerve fibre layer; PPV: pars plana vitrectomy).

During the ocular clinical examination of the treated eyes prior to enucleation, all the porcine lenses had remained clear at 30 days into the postoperative period. On indirect ophthalmoscopy, the retina remained attached in all eyes with no visible changes to the neuro-retina, retinal vasculature, retinal pigment epithelium (RPE) or optic nerve head (ONH). One eye each treated with the HV and GV (Eye No.5 and Eye No.13 respectively) showed some evidence of RPE atrophy likely attributable to an inadvertent retinal touch with the needle during surgery. As per Table 2, decreased RPE pigmentation was a histological feature seen in eyes 3 and 7 whilst “atrophy” was described at clinical examination in eyes 5 and 13. Artefacts, such as cytoplasmic vacuolation in the GCL or disruption in the INL and NFL were observed in most eyes, including the control eyes (Fig 1).

**Fig 2:**
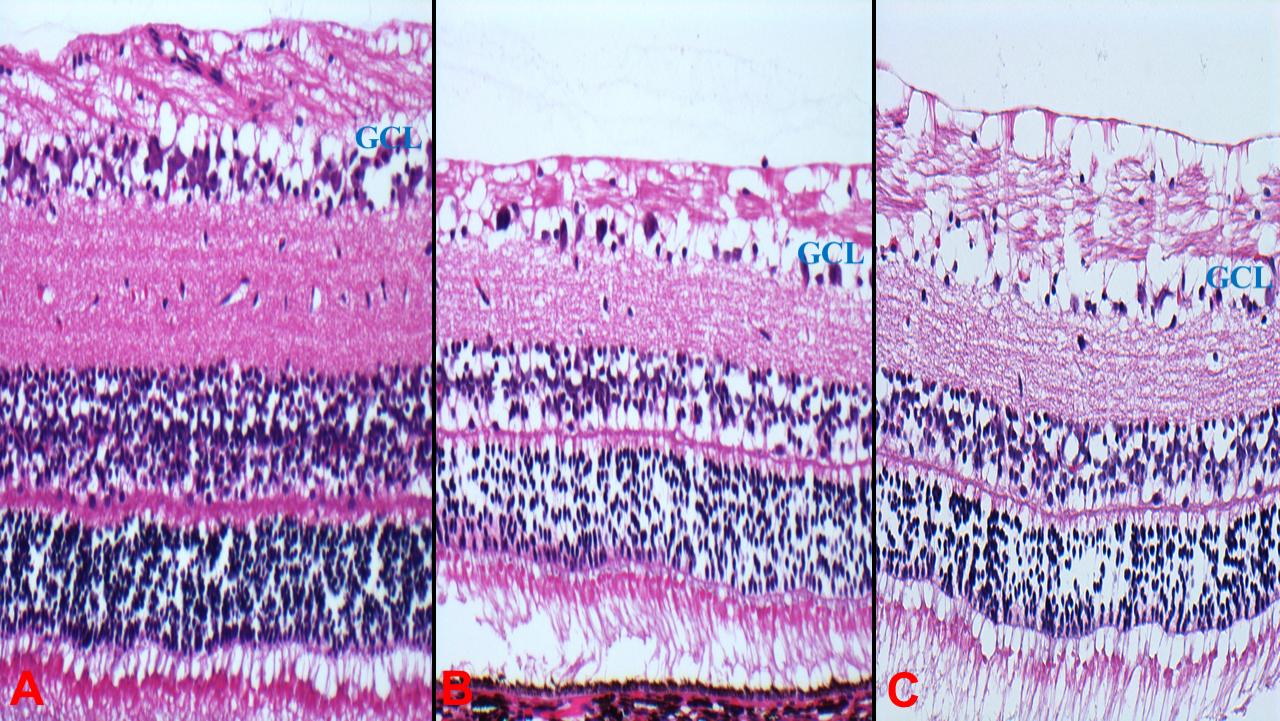
Haematoxylin-eosin stained horizontal retinal sections from porcine enucleated eyes in Eye No.15 at different stroke lengths with HV PPV. (A) Nasal macula (10μm) (B) Temporal macula (20μm). There appeared to be more cytoplasmic vacuolation along GCL with higher stroke length (B). However, similar cytoplasmic vacuolation changes along the GCL were noted in the control eye of same pig (C). (GCL: ganglion cell layer; HV: hypersonic vitrector; PPV: pars plana vitrectomy).

For Eye No.15, used for stroke length testing, no anomalies were noted pre-enucleation. Microscopic examination of the retina showed mild cytoplasmic vacuolation along the ganglion cell layer (GCL) nasally and moderate vacuolation in the temporal GCL and inner nuclear layer (INL). The temporal macular area was tested with a higher stroke length (20 μm) than the nasal macular area (10 μm) whereas superior and inferior to the ONH was tested with 30 μm and 40 μm respectively (Fig 2).

Posterior vitreous detachment (PVD) inducement was successful in two of the three HV animal subject eyes (Eye No.5 and No.11). PVD inducement could not be attempted in Eye No.7 because there was no remaining vitreous attached to the retina following core vitrectomy. This was assessed by visualizing the vitreous using intravitreal triamcinolone acetonide.

On a subjective basis, the performance of the HV was assessed by the surgeon (PES) to be equivalent to the GV (Table 3). One exception was the HV with the 175 μm round port needle which was judged to be substandard as compared to the pneumatic cutter for peripheral vitreous removal. Along with subjective efficiency ratings, the time to perform the various surgical segments for each treated eye was recorded intraoperatively and shown in Table 4.

**Table 3:** Surgeon subjective ratings of 23G HV efficiency compared to the 23G GV.

			Surgeon Subjective Rating of HV Efficiency				
Eye No.	HV Port Size	Surgical Segment	^a^Exceeds Standards	^a^Meets Standards	^a^Somewhat Below Standards	^a^Far Below Standards	N/A
3	175 μm	Core vitrectomy		X			
		PVD inducement					X
		Posterior vitreous removal		X			
		Peripheral vitreous removal				X	
5	225 μm	Core vitrectomy		X			
		PVD inducement		X			
		Posterior vitreous removal		X			
		Peripheral vitreous removal		X			
7	225 μm	Core vitrectomy		X			
		PVD inducement		X			
		Posterior vitreous removal					X
		Peripheral vitreous removal					X
11	225 μm	Core vitrectomy		X			
		PVD inducement		X			
		Posterior vitreous removal		X			
		Peripheral vitreous removal		X			

^a^Standard = Performance of the 23G GV for the same procedure.

N/A: Not available

**Table 4:** Observed time to perform core vitrectomy and full PPV procedure.

				Performance Time (minutes)	
Vitrectomy Type	Eye No.	Needle Port Size	PVD Induced	Core vitrectomy	Full PPV Procedure
**23G HV**	3	175 μm	No^a^	4.53	8.33
	5	225 μm	Yes	3.43	6.13
	7		No^b^	1.61	8.73
	11		Yes	1.75	9.43
	**Mean time**			2.27	8.10
**23G GV**	9	N/A	Yes	1.10	3.88
	13	N/A	No	1.68	5.48
	**Mean time**			1.39	4.68

^a^PVD was not induced as part of the surgical protocol

^b^PVD was not completed because there was no remaining vitreous attached to the retina after core vitrectomy.

GV: Guillotine vitrector; HV: Hypersonic vitrector; N/A: Not applicable

## Discussion

High-power, low-frequency US devices have many clinical applications, in surgical instruments for tissue cutting, ablation, fragmentation and removal. The literature on the tissue ablation and damage mechanism of these instruments is poorly understand and is predominantly subjective and based on clinical observation [4–11]. The described US tissue damage includes global biomechanical properties alteration, histo-morphological changes, protein denaturation and tissue necrosis [10–14]. In our previous work, comparison between the control eyes, porcine and human cadaveric eyes that had PPV with GV or HV had histopathological findings primarily at the inner retinal layers, with some degree of vacuolization and fragmentation in the NFL and GCL, as well as internal limiting membrane (ILM) separation [2]. These changes are similar in this study 30 days following PPV with either the HV or GV. The observed impacts from both vitrectomy systems are not clearly distinguishable from one another and do not differ conclusively from the control samples. Artefacts, such as cytoplasmic vacuolation in the GCL or disruption in the INL and NFL were observed in most eyes, including the control eyes. This vacuolation process has been described as a ‘cavitation’ phenomenon which occurs at intracellular or extracellular levels causing cell fragmentation and cell destruction or causing inefficient coupling with energy dissipation with surrounding fluid but no cellular fragmentation [10]. Compared to previous studies [11–14], our study did not show any extensive cell fragmentation in all samples and no major RPE disturbance other than due to iatrogenic retinal touch. We have also addressed the limitations from our previous study such as using live porcine eyes and observing their delayed effects after 30 days instead of cadaveric eyes to limit normal post-mortem changes as well as assessing the long-term effects associated with the HV.

When the HV was operated at stroke length settings ranging from 10–40μm in close proximity to the retina, there appears to be more retinal impact with increasing stroke length settings (Fig 2). These results must be interpreted cautiously as vacuolation of the GCL and INL were a common finding for both treated and control eyes, and may not be directly attributable to stroke length settings. These findings may also be related to the extent of any underlying retinal detachment from the tissue fixation procedure or time to fixation. These observed retinal changes (vacuolization and fragmentation) have also been described in normal post-mortem changes [15] or to excessive amount of suction during vitrectomy procedures [16]. Therefore, no firm conclusions can be drawn in this study regarding the impact of varying stroke length settings near the retina.

Regarding the efficiency of the HV compared to the GV, the subjective ratings from the surgeon suggest the devices are comparable. Performance times gathered during this study suggest that the HV may not be as efficient for core vitrectomy or for a full PPV procedure. However, these results should be interpreted very cautiously as the sample sizes are very small for any statistical conclusions. Furthermore, during the procedures with the HV, experimentation was required to optimize or identify effective settings such as the infusion pressure and stroke length. The time to conduct this additional experimentation is reflected in the performance times, and would not be a factor once the HV technology is established and optimized settings are well-understood. Besides that, the unfamiliarity of the new system and period of adjustment may have affected the performance time and satisfaction rating of the surgeon.

Although we have addressed most of the limitations from our previous study [2], one of the main limitations in this study is that we have not performed electro-diagnostic tests such as electroretinography or visual evoked potentials to assess the functionality of the retina or optic nerve head cells following the HV. As most of the changes are limited to the inner retinal layers rather than the RPE layers, we can only assume that most of the functionality of the retina layers are intact without performing the electrodiagnostic tests. Due to the general consistency in the histological findings for the HV, GV and control samples, it is difficult to distinguish if these findings were the direct result of the hand-piece type, PPV procedure, the insertion of the ESA devices, or processing of the samples. However, as the results are similar to our previous study on the cadaveric eyes and non-delay live animal surgeries, we could conclude that at 30 days into the postoperative period, there seemed to be no subjective safety or efficacy issues attributable to the sole use of the HV but this study is limited by the lack of an objective assessment such as the use of electrodiagnostic tests to confirm the safety of the HV on the retinal tissues. Further studies in animal studies and live human eyes with the HV using the electrodiagnostic test will help transition its use to commercial use in the future.
